# Florid Diabetic Retinopathy in a Young Patient

**Published:** 2012-01

**Authors:** Syed Shoeb Ahmad, Shuaibah Abdul Ghani

**Affiliations:** Ophthalmology Department, Queen Elizabeth Hospital, Kota Kinabalu, Malaysia

In May 2008, a 21-year-old single, non-obese, diabetic lady was referred to our clinic for routine eye examinations. The patient had been diagnosed with diabetes mellitus three months earlier and was receiving 100 milligrams of metformin daily and 80 milligrams of gliclazide twice daily orally, consistently maintaining her blood glucose levels between 72–90 mg/dl. She had a positive family history of diabetes mellitus. No related comorbidities were reported in her nephrology and rheumatology consultations and she had no visual complaints such as floaters, blurred vision, metamorphopsia or photophobia.

Ocular examination revealed bilateral best corrected visual acuity (BCVA) of 6/18, and normal anterior segments without neovascularization on the iris or in the angles. However, fundus examination showed florid neovascularization of the disc (NVD) in both eyes (Fig. 1). The vessels were profuse and extended all around the discs. The condition was termed “coralliform” NVD for its resemblance to sea corals. Other areas of neovascularization (NVE) were also seen in both eyes. The macular region was edematous bilaterally with a few hard exudates. Optical coherence tomography confirmed the presence of macular edema (Fig. 2).

The condition was explained to the patient and informed consent was obtained for bilateral panretinal laser photocoagulation (PRP) and grid laser photocoagulation. An initial grid laser treatment followed by multiple sessions of PRP was performed in both eyes (Fig. 3). Despite two sessions of grid laser, the macular edema persisted. She refused receiving intraocular injections, therefore bilateral orbital floor injections of triamcinolone acetonide (20 milligrams) were administered twice within a one month interval. Subsequently, the macular edema resolved.

The last examination in March 2011 revealed BCVA of 6/10 in both eyes and significant regression of NVD and NVE. There was no macular edema (Fig. 4) and the patient was satisfied with her vision.

## DISCUSSION

Florid diabetic retinopathy (FDR) is characterized by proliferative diabetic retinopathy in a young patient, with predilection for females, bilateral affliction and a rapid course which may lead to blindness in a short time. The condition is usually exacerbated by poor metabolic control. This condition is assumed to be secondary to acute ischemia. High blood levels of growth hormone have also been implicated in its causation. An important pathophysiologic mechanism appears to be diffuse retinal ischemia with widespread blood-retinal barrier breakdown.^1^ It has been postulated that acute hypoglycemic episodes together with transient increases in serum levels of insulin-like growth factor 1 (IGF-1) and vitreous levels of vascular endothelial growth factor (VEGF) contribute to the development of FDR.^2^ Despite improved management techniques, the prognosis of FDR remains guarded. Seyer-Hansen^3^ reported a 22-year-old man who developed total blindness within 30 months of the diagnosis of diabetes mellitus. In a long term study on FDR by Lettanzio^1^, patients were categorized into two groups according to baseline retinal condition: group 1 included eyes amenable to laser photocoagulation and when necessary, to subsequent vitreoretinal surgery; whereas group 2 eyes consisted of eyes with more advanced diabetic retinopathy, directly undergoing vitreoretinal surgery and laser photocoagulation. The patients were followed for 54 months; 75% of eyes in group 1 had a favorable anatomical outcome with mean final visual acuity of 0.47. In group 2, diabetic retinopathy progressed in 68% of eyes and mean final visual acuity was 0.14. The latter group had 6 times higher rates of blindness as compared to group 1 (31% versus 5%). The authors concluded that early diagnosis and treatment can halt the progression of FDR in three out of four cases.

Kohner et al^4^ reported a group of 34 patients with FDR. The patients were divided into no treatment, PRP, or pituitary ablation. After one year, of the 11 eyes which had undergone PRP, 6 were blind while 5 had good vision; by the end of the second year, only 3 eyes had good vision and the rest were blind. Out of a total of 20 patients who underwent pituitary ablation, at the end of the first year only 3 eyes were blind, while after 5 years 12 of 17 eyes could still see and only 2 more had become blind. The authors claimed that in this rare form of retinopathy, pituitary ablation remains the treatment of choice if vision is to be maintained. Valone and McMeel^5^ also reported better vision preservation in patients undergoing pituitary ablation, as compared to untreated subjects. An earlier report on 9 patients treated with pituitary ablation or laser photocoagulation noted regression of FDR in both groups.^6^ FDR was also reported to regress with an insulin-infusion device^7^ and also with continuous subcutaneous insulin infusion^8^. Casati et al^9^ reported a 17-year-old girl with Donahue syndrome and FDR. Her 5 year follow up after PRP revealed satisfactory regression of NVD and maintenance of good visual acuity. Nevertheless, diabetic retinopathy has been known to deteriorate rapidly after institution of strict metabolic control.^10^,^11^

The patient described herein is a typical case of FDR; a 21-year-old diabetic lady with severe bilateral proliferative diabetic retinopathy at presentation. She demonstrated a favorable response to aggressive PRP. The macular edema also resolved with a judicious combination of grid laser photocoagulation and orbital floor triamcinolone acetonide injection. Bandello et al^12^ have reported a case of FDR, where intravitreal triamcinolone acetonide (IVTA) followed by PRP was done for one eye and the other eye underwent PRP alone. The authors reported greater reduction in retinal thickening and fluorescein leakage from retinal new vessels in the eye which had been given IVTA.

In summary, FDR is an aggressive presentation of diabetic retinopathy; therefore all young diabetics should be screened as soon as possible. These patients need to be closely monitored to identify the condition at its inception. Once FDR develops, the patient needs to be treated aggressively with laser photocoagulation and possibly vitrectomy. However, FDR still remains an unfortunate presentation in young diabetics and portends a guarded prognosis.

## Figures and Tables

**Figure 1.**
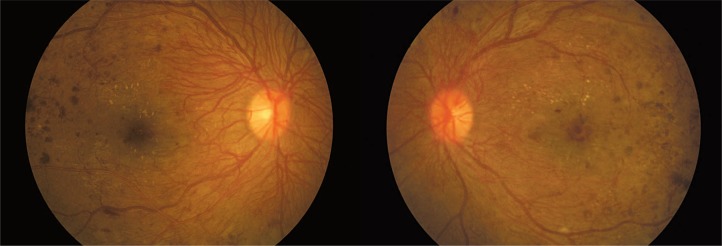
Coralliform NVD in the right eye at initial presentation (left image); NVD and macular edema in the left eye at initial presentation (right image).

**Figure 2.**
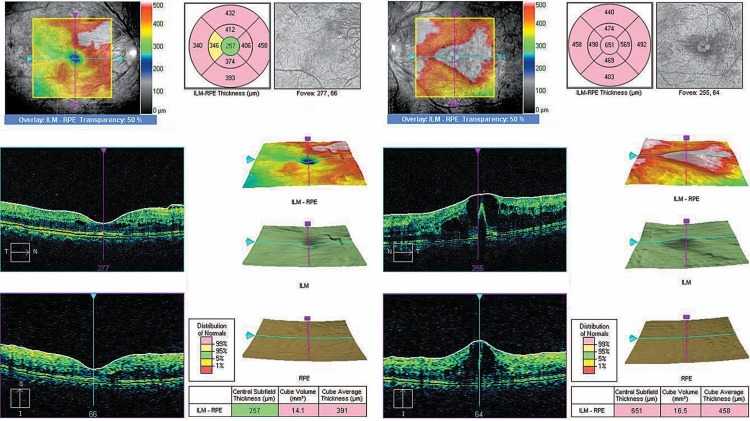
Optical coherence tomography at presentation in the right and left eyes (left and right images, respectively).

**Figure 3.**
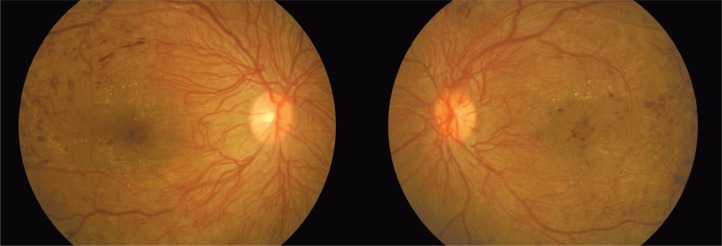
Right eye after laser photocoagulation (left image); left eye after initiation of laser photocoagulation (right image).

**Figure 4.**
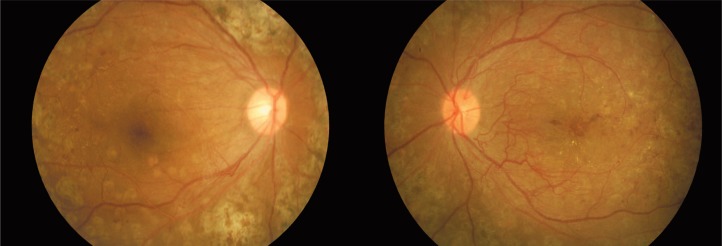
Fundus appearance in the right (left image) and left eye (right image) at final follow-up.
